# Expression of genes related to the hypothalamic-pituitary-adrenal axis in murine fetal lungs in late gestation

**DOI:** 10.1186/1477-7827-8-134

**Published:** 2010-11-04

**Authors:** Marc Simard, Mélissa Côté, Pierre R Provost, Yves Tremblay

**Affiliations:** 1 Reproduction, Perinatal Health, and Child Health, CHUQ Research Center, Québec City, Québec, Canada; 2 Centre de Recherche en Biologie de la Reproduction (CRBR), Laval University, Québec City, Québec, Canada; 3 Department of Obstetrics and Gynecology, Faculty of Medicine, Laval University, Québec City, Québec, Canada

## Abstract

**Background:**

Lung maturation is modulated by several factors, including glucocorticoids. Expression of hypothalamic-pituitary-adrenal (HPA) axis-related components, with proposed or described local regulatory systems analogous to the HPA axis, was reported in peripheral tissues. Here, HPA axis-related genes were studied in the mouse developing lung during a period overlapping the surge of surfactant production.

**Methods:**

Expression of genes encoding for corticotropin-releasing hormone (CRH), CRH receptors (CRHR) 1 and 2beta, CRH-binding protein, proopiomelanocortin (POMC), melanocortin receptor 2 (MC2R), and glucocorticoid receptor was quantified by real-time PCR and localized by in situ hydridization in fetal lungs at gestational days (GD) 15.5, 16.5, and 17.5, and was also quantified in primary mesenchymal- and epithelial cell-enriched cultures. In addition, the capability of CRH and adrenocorticotropic hormone (ACTH) to stimulate pulmonary expression of enzymes involved in the adrenal pathway of glucocorticoid synthesis was addressed, as well as the glucocorticoid production by fetal lung explants.

**Results:**

We report that all the studied genes are expressed in fetal lungs according to different patterns. On GD 15.5, Mc2r showed peaks in expression in samples that have previously presented high mRNA levels for glucocorticoid synthesizing enzymes, including 11beta-hydroxylase (Cyp11b1). Crhr1 mRNA co-localized with Pomc mRNA in cells surrounding the proximal epithelium on GD 15.5 and 16.5. A transition in expression sites toward distal epithelial cells was observed between GD 15.5 and 17.5 for all the studied genes. CRH or ACTH stimulation of genes involved in the adrenal pathway of glucocorticoid synthesis was not observed in lung explants on GD 15.5, whereas CRH significantly increased expression of 21-hydroxylase (Cyp21a1) on GD 17.5. A deoxycorticosterone production by fetal lung explants was observed.

**Conclusions:**

Temporal and spatial modulations of expression of HPA axis-related genes in late gestation are consistent with roles for these genes in lung development. Our data are likely to lead to valuable insights in relation to lung diseases originating from lung immaturity.

## Background

Corticotropin-releasing hormone (CRH) and adrenocorticotropic hormone (ACTH) are classically involved in the modulation of the hypothalamic-pituitary-adrenal (HPA) axis, leading to secretion of glucocorticoids by the adrenal glands [1,2]. CRH also has a role in regulating neuroendocrine functions, reproduction, and immune functions [3]. The proopiomelanocortin (*Pomc*) gene encodes for several peptides with various roles, including ACTH, with highly tissue-specific regulation and processing [4,5]. During gestation, glucocorticoids of adrenal origin are involved in the maturation of many fetal organ systems, including the lung [6,7]. Glucocorticoids are administered to pregnant women at risk of premature delivery to accelerate fetal lung maturation and to reduce the occurrence and severity of respiratory distress syndrome (RDS) [8]. Their importance in fetal lung development was highlighted by CRH-null and glucocorticoid receptor (GR)-null mouse models, in which mice display an abnormal lung phenotype and die at birth from respiratory failure [9,10].

Limited information is available on expression of CRH, ACTH, and other HPA axis-related components in the fetal lung. *Crh *mRNA was localized in fetal mouse lungs around branching bronchioles [11], CRH was detected in baboon fetal lungs [12], and *POMC *mRNA was detected in ovine fetal lungs [13]. A direct effect of CRH was demonstrated on differentiation and proliferation of type II pneumocytes in baboon fetal lung explants [14]. CRH receptor type 1 (CRHR1), which is associated with pituitary ACTH secretion, was detected at the mRNA level in baboon fetal lungs [14]. In addition, the melanocortin 2 receptor (MC2R), which leads to glucocorticoid production in adrenal glands following binding to its only known ligand ACTH [5], was detected in developing mouse lungs [15].

Expression of *Crh *and *Pomc *was shown in several peripheral tissues [5,16], including thymus [17,18] and skin [19]. In the latter, CRH/ACTH signaling was reported, leading to local glucocorticoid production. Brain, cardiovascular system, and thymus were also identified as production sites of glucocorticoids involving an adrenal-like enzymatic pathway [20,21]. However, the implication of CRH and ACTH in these glucocorticoid productions remains to be defined. Our laboratory previously reported that genes involved in the adrenal pathway of glucocorticoid synthesis, namely steroidogenic acute regulatory protein (*Star*), cytochrome P450 side chain cleavage (*Cyp11a1*), 3β-hydroxysteroid dehydrogenase (*Hsd3b1*), 21-hydroxylase (*Cyp21a1*), and 11β-hydroxylase (*Cyp11b1*), are transiently co-expressed at high levels in the fetal mouse lung on gestational day (GD) 15 [22]. Whether this co-expression is modulated by pituitary ACTH, by an autocrine/paracrine mechanism involving a local HPA axis-like pathway, or by a CRH/ACTH-independent mechanism, remains to be determined.

Based on data showing 1) the expression of HPA axis-related genes in some peripheral tissues, 2) the existence of a functional HPA axis-like pathway leading to glucocorticoid production in the skin, and 3) the transient expression of genes involved in the adrenal pathway of glucocorticoid synthesis in the murine fetal lung, we hypothesized that HPA axis-related genes could be developmentally regulated in the murine fetal lung and that CRH and/or ACTH could be involved in the regulation of fetal pulmonary expression of enzymes leading to glucocorticoid production. In order to question their involvement in lung development, HPA axis-related genes were studied in the developing lung where their expression profiles as well as their sites of expression were investigated. For that purpose, *Crh*, *Crhr1*, *Crhr2b*, *Crhbp*, *Pomc*, *Mc2r*, and *Nr3c1 *(GR) were studied by QPCR and *in situ *hybridization in male and female fetal mouse lungs on GD 15.5, 16.5, and 17.5. This gestation window overlaps the surge of surfactant production occurring on GD 17.5 [23-25]. Immunoreactive ACTH (iACTH) was also detected by immunohistochemistry. The existence of a male disadvantage in the occurrence of RDS [26], as well as the proposed transient delay for one sex in the expression of genes involved in glucocorticoid synthesis in the developing lung [22], warranted the consideration of sex. Several adult tissues including lung, spleen, thymus, brain, and adrenal gland were included in QPCR experiments for comparison. Messenger RNA levels were also measured in epithelial cell-enriched and fibroblast-enriched primary cultures originating from mouse fetal lungs to further characterize expression profiles of the target genes. In addition, fetal lung explants were incubated in the presence of CRH or ACTH to evaluate the capability of these hormones to stimulate the expression of *Pomc*, *Star*, *Hsd3b1*, *Cyp21a1*, and *Cyp11b1*, and the glucocorticoid production by fetal lung explants was addressed.

## Methods

### Animals, housing, and fetal tissue preparation

Protocol was approved by the Animal Care and Use Committee and the Institutional Review Board of the CHUQ Research Ctr (protocol no. 2008-071). BALB/c mice (*Mus musculus*) aged 63-70 days and certified pathogen-free were purchased (Charles River Laboratories, St-Constant, QC, Canada) and housed in a room maintained at 22°C, 50% relative humidity and on a 12-hours cycle (07:15-19:15 hours) of fluorescent lighting (300 Lux). Global 18% Protein Rodent Diet (Teklad, Montréal, QC, Canada) and tap water were provided *ad libitum*. New animals were acclimatized to these conditions for 7-14 days prior to be mated. Fetuses were obtained from overnight periods of mating. The day of vaginal plug was considered as GD 0.5. Pregnant females were sacrificed by exposure to a CO_2 _atmosphere. Fetal sex was visually established and confirmed in some cases by PCR amplification of *Sry *as previously described [27]. Lungs were rinsed thoroughly in phosphate buffered saline (PBS) and snap-frozen before storage at -80°C, or put in 4% w/v paraformaldehyde for 48 hours and then paraffin-embedded. Slices of 5 μm were prepared for *in situ *hybridization and immunohistochemistry experiments. A minimum of three litters, including females and males, were studied on GD 15.5, 16.5, and 17.5.

### RNA probes

RNA probe templates were prepared from mouse lung and brain cDNAs, as previously described [28]. Briefly, a specific fragment of each studied gene was amplified and inserted into pGEM-4Z (Promega Corp., Madison, WI, USA). Specific primer pairs used for PCR amplifications are presented in Table 1. Antisense and sense RNA probes were synthesized using DIG-UTP substrate (Roche Diagnostics, Laval, QC, Canada) from PCR products amplified with specific primers for SP6 and T7 promoters: GGATTTAGGTGACACTATAGAATA and TAATACGACTCACTATAGGGAGAC, respectively.

**Table 1:** Oligonucleotides and experimental parameters for QPCR, and oligonucleotides for *in situ *hydridization probe synthesis, for experiments in mouse fetal lungs

**Genes**^**a **^**(NCBI)**	**Oligonucleotides**^**b**^	Amplicon length (bp)	**QPCR conditions**^**c**^		
	QPCR: forward/reverse RNA probes: forward/reverse	Probe position	Ann. T°C	Acq. T°C	# of cycles
	*Target genes*				
*Pomc *(NM_008895)	CATTAGGCTTGGAGCAGGTC/TCTTGATGATGGCGTTCTTG	174	66	88	45
	ACGTGGAAGATGCCGAGATTCTGCTACAGTC/CAGGAACAGCAGCAGTGC	141-433			
*Mc2r *(NM_008560)	CCTCTCTTTGCTGGGCTCTA/GCACCCTTCATGTTGGTTCT	321	63	83	40
	CCTCTCTTTGCTGGGCTCTA/GCACCCTTCATGTTGGTTCT	589-909			
*Crh *(NM_205769)	CCTGGGGAATCTCAACAGAA/AACACGCGGAAAAAGTTAGC	120	62	89	45
	TCCGCATGGGTGAAGAATAC/GGTGGAAGGTGAGATCCAGA	347-649			
*Crhbp *(NM_198408)	TCTTACCAGAAGGAGCATCAG/GTGTCCGAGGGTAAGATCAG	241	63	81	45
	CGGGGATTTCCTGAAGGTAT/CTGCAGTTTTGGTGCTGGTA	469-768			
*Crhr1 *(NM_007762)	CCAGGATCAGCAGTGTGAGA/TGTTGTAGCGGACACCGTAG	161	64	86	43
	CCAGGATCAGCAGTGTGAGA/AGTGGCCCAGGTAGTTGATG	265-372			
*Crhr2b *(NM_009953)	CAGGCCAGGCACCCCAGGAC/GGAACCACGCGATGTTTCTCAG	477	68	85	43
	GGCTTTACCTTGGTGGGTAG/TGAAAAATTCCCAGTTGTGGTC	162-492			
*Nr3c1 *(NM_008173)	GGGGAATTCACAGACTTTCGGCTTCTGGA/GGGAAGCTTCTGACCTCCAAGGACTCTCG	297	63	83	40
	ACCTGGATGACCAAATGACC/AGGAGAGAAGCAGTAAGGTTTTCA	1821-2078			
*Mrap *(NM_029844)	TTCGTGGTGCTCCTCTTTCT/TCCTGGCTCCTCTGTTGTCT	162	63	85	43
*Cyp21a1 *(NM_009995)	CCTTGCCCCATCGTGCAACTA/TGGAGGCAGCAGAGTGAAGG	278	67	85	43
*Vim *(NM_011701)	GATCAGCTCACCAACGACAA/AAGACGTGCCAGAGAAGCAT	170	62	84	40
*Krt18 *(NM_010664)	GTGGAATCCAGACCGAGAAA/GTTCTTCCTTGAGTGCCTCG	406	58	84	40
	*Housekeeping genes*				
*Gapdh *(NM_008084)	GTCGGTGTGAACGGATTTG/AAGATGGTGATGGGCTTCC	215	61	84	40
*Hprt1 *(NM_013556)	AGTCCCAGCGTCGTGATTAG/AATCCAGCAGGTCAGCAAAG	228	64	82	40
*Sdha *(BC031849)	ACACAGACCTGGTGGAGACC/CAAACGGCTTCTTCTGCTGT	179	59	84	45

^a^*Pomc*, proopiomelanocortin; *Mc2r*, melanocortin receptor 2 (ACTH receptor); *Crh*, corticotropin releasing hormone; *Crhbp*, corticotropin releasing hormone binding protein; *Crhr1*, corticotropin releasing hormone receptor 1; *Crhr2b*, corticotropin releasing hormone receptor 2β; *Nr3c1*, nuclear receptor subfamily 3, group C, member 1 (glucocorticoid receptor); *Mrap*, melanocortin2 receptor accessory protein; *Cyp21a1*, 21-hydroxylase; *Vim*, vimentin; *Krt18*, keratin 18; *Gapdh*, glyceraldehyde-3-phosphate dehydrogenase; *Hprt1*, hypoxanthine phosphoribosyltransferase 1; *Sdha*, succinate dehydrogenase complex, subunit A, flavoprotein.

^b^Primer pairs used in QPCR experiments are presented. Primer pairs used to amplify and clone specific fragments to be used in RNA probe synthesis are underlined. Primer pairs for cloning contain sequences for restriction endonucleases: forward primers, GGGGAATTC; reverse primers, GGGAAGCTT.

^c^Ann. T°C = annealing temperature; Acq. T°C = acquisition (fluorescence intensity reading) temperature.

### In situ hybridization and immunohistochemistry

*In situ *hybridization was performed as previously described [28]. Hybridization with specific RNA probes (5 ng/ul) was performed overnight at 42°C. Tissue sections were incubated in substrate solution [28] three hours for *Crh*, *Pomc*, and *Nr3c1 *or overnight for *Crhr1*, *Crhr2b*, *Crhbp*, and *Mc2r*. Immunohistochemistry was performed as previously described [28]. The anti-immunoreactive ACTH (iACTH) antibody (rabbit anti-mouse; kindly provided by Dr Georges Pelletier, CHUQ Research Ctr, [29]) and a rabbit IgG preparation (Vector Laboratories Inc, Burlington, ON, Canada) were incubated overnight at 4°C. Microscopy was performed with a Zeiss Axioskop 2 Plus microscope (Carl Zeiss Canada, Toronto, ON, Canada) equipped with Zeiss Plan-Neofluar objectives. Images were acquired with a QImaging Retiga 2000R camera (QImaging, Surrey, BC, Canada) using the Image-Pro Plus 5.1 software (Media Cybernetics Inc, Bethesda, MD, USA).

### Quantitative real-time PCR and normalization with multiple housekeeping genes

RNA extraction and cDNA synthesis [30], and quantitative real-time PCR [31], were performed as described previously. The analyzed genes and quantitative real-time PCR conditions are presented in Table 1, except for *Star*, *Hsd3b1*, and *Cyp11b1 *[22]. After enzyme activation (10 min, 95°C), PCR cycles were performed: 5 sec, denaturation 95°C; 5 sec, annealing (see Table 1 for temperature); 20 sec, elongation 72°C; 5 sec, temperature of fluorescence intensity reading (see Table 1). Expression stability of three housekeeping genes (*Gapdh*, *Hprt1*, *Sdha*) was assessed based on the approach proposed by Vandesompele et al [32]. Then, normalization factors were calculated from expression levels of the three housekeeping genes.

### Preparation of fibroblast- and epithelial cell-enriched cultures from fetal mouse lungs

Fetal lungs were removed, rinsed in PBS, cut into pieces of approximately 2 mm^3^, and extensively rinsed again in PBS. Digestion was made in Hank's buffered saline solution (HBSS) (Multicell, Wisent Inc., St-Bruno, QC, Canada) containing 0.25% w/v trypsin (Multicell), 0.17% w/v collagenase (Sigma, St. Louis, MO, USA), DNAse I (25 μg/ml) (Sigma), penicillin (50 iu)/streptomycin (50 μg/ml) (Multicell), and gentamycin (5 μg/ml) (Sigma) at 37°C for 30 minutes under agitation. Digestion was stopped by addition of Dulbecco's modified eagle's medium (DMEM) (Multicell) containing 20% v/v fetal bovine serum (FBS) (Sigma). Residual tissue debris were discarded by 1 × *g *sedimentation and then, supernatants were centrifuged 7 min at 100 × *g*. Cell pellets were resuspended in DMEM containing 20% v/v FBS and penicillin (50 iu)/streptomycin (50 μg/ml). Cells were incubated for one hour at 37°C with 5% CO_2 _to allow fibroblast adhesion. Then, culture media were removed and the fibroblast adhesion step was repeated twice. The epithelial cell-enriched fractions (non-adherent cells) were collected and filtered through a sterile 45 μm cell strainer (VWR International, Montréal, QC, Canada), centrifuged 7 min at 100 × *g *and then plated in 12-well plates. Cell cultures were kept 12 hours at 37°C under 5% CO_2 _atmosphere. Cell phenotypes were visually confirmed before harvesting. Epithelial cell-enriched cultures were then homogenized in TRI Reagent (Molecular Research Center, Cincinnati, OH, USA) for RNA extraction. Fibroblast-enriched cultures were filtered through a sterile 45 μm cell strainer before RNA extraction. Three separate cell enrichment experiments with 2-3 litters each were done at GD15.5 (total n = 44 fetal lungs) and four were done at GD17.5 (total n = 42 fetal lungs). Given the low number of epithelial cells recovered at GD15.5, RNA extracts were pooled before cDNA preparation. At GD17.5, enough epithelial cells for cDNA synthesis were recovered in 2 out of 4 experiments.

### Incubation of lung explants with CRH or ACTH

GD 15.5 and 17.5 fetal lungs were removed, rinsed in PBS, and incubated 8 h in DMEM containing penicillin (50 iu)/streptomycin (50 μg/ml) and 10^-7 ^M CRH (C3042, Sigma) or 10^-7 ^M ACTH (Cortrosyn, Amphastar Pharmaceuticals Inc., Rancho Cucamonga, CA, USA) at 37°C under gentle agitation. Three and four litters were analyzed at GD 15.5 and 17.5, respectively, with 1 to 3 fetal lungs per condition (control, CRH, and ACTH) for each litter. Explants were then homogenized in TRI reagent and stored at -80°C until RNA extraction.

### Steroidogenic activity measures

Deoxycorticosterone (DOC) production was measured in control and CRH-treated-GD17.5 lung explants. Five litters were used, with 2-3 fetal lungs per litter per condition. The explants were incubated in 1 mL of DMEM containing pen/strep a first 3 h with 2 × 10^-7 ^M CRH or without, then 5 h with 2 × 10^-7 ^M CRH or without in the presence of [1,2,6,7-^3^H(N)]progesterone at 58 nM (6 μCi/well) (Perkin Elmer, Boston, MA) and unlabeled DOC (Steraloids, Wilton, NH) at 10^-5 ^M. Unlabeled DOC was added to reduce metabolization of tritiated DOC. Steroids were extracted from culture media with chlorobutane, and resolved by thin layer chromatography (toluene 78: acetone 20: methanol 2). Tritiated standards of progesterone, DOC, and corticosterone were included. Revelation of the tritiated products and quantification were performed using a Storm apparatus (Molecular Dynamics, Inc., Sunnyvale, CA). Data are expressed as deoxycorticosterone radioactivity count/total radioactivity count/mg tissue.

### Statistical analysis

Statistical analyses were performed using GraphPad Prism 5.01 software (GraphPad Software, La Jolla, CA, USA). Two-way ANOVA with randomized-block design was used for QPCR experiments on total fetal lung extracts, where male and female values were matched. One-way ANOVA with randomized-block design followed by a Tukey's test was used for experiments with lung explants incubated with CRH or ACTH, where samples from the same litter were matched. Paired t-test was used to analyze data of deoxycorticosterone quantification, where mean control samples and mean treated samples from each litter were paired. When normality of data could not be assumed following a normality test in GraphPad Prism, logN transformation was performed. A difference with a *P *value of less than 0.05 was considered as significant.

### Results

### Expression levels of HPA axis-related genes in male and female fetal mouse lungs

**Figure 1:**
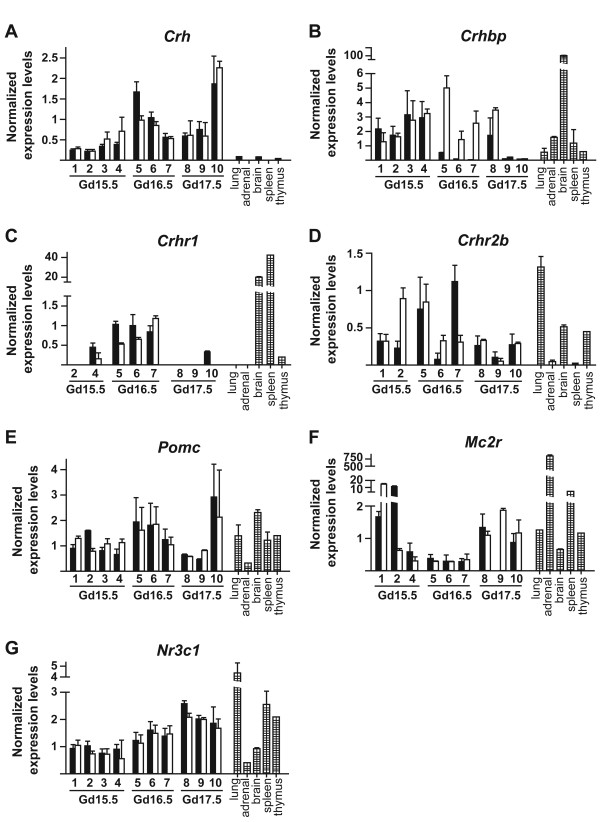
**Relative mRNA expression levels of HPA axis-related genes in mouse fetal lungs**. *Crh *(A), *Crhbp *(B), *Crhr1 *(C), *Crhr2b *(D), *Pomc *(E), *Mc2r *(F), and *Nr3c1 *(G) were quantified by QPCR in male and female total fetal lung extracts from several litters on GD 15.5, 16.5, and 17.5. For each litter, the black bar represents the male pool and the white bar represents the female pool. Dashed bars represent adult tissues. Number of animals in each litter (litter #: number of males (M), number of females (F)): #1: 2 M, 2 F; #2: 4 M, 5 F; #3: 4 M, 3 F; #4: 2 M, 3 F; #5: 3 M, 6 F; #6: 4 M, 3 F; #7: 1 M, 2 F; #8: 4 M, 4 F; #9: 3 M, 4 F; #10: 3 M, 1 F. Each value (± SD) was normalized using a factor generated from expression values of three housekeeping genes, namely *Hprt1*, *Sdha*, and *Gapdh*. ANOVA was performed to evaluate the effect of time and sex. A trend in increase in *Crh *mRNA levels was observed according to gestation time (p = 0.053) (A), a significant interaction between time and sex (p = 0.019), as well as a significant impact of sex (p = 0.017), were observed for *Crhbp *mRNA levels (B), and a significant increase in *Nr3c1 *mRNA levels was observed over time (p = 0.0003) (G).

The gene expression profiles of *Crh*, *Crhbp*, *Crhr1*, *Crhr2b*, *Pomc*, *Mc2r*, and *Nr3c1 *were determined in male and female fetal lung pools from several mouse litters collected on GD 15.5, 16.5, and 17.5 (Figure 1). Several mature tissues were included for reference. To give estimates of raw mRNA levels in fetal lungs, mean crossing point values are presented in Table 2.

**Table 2:** Mean crossing point (cp) values of the amplified genes in mouse fetal lungs

	Target genes								Housekeeping genes		
	*Crh*	*Crhbp*	*Crhr1*	*Crhr2b*	*Pomc*	*Mc2r*	*Nr3c1*	*Mrap*	*Hprt1*	*Sdha*	*Gapdh*
Mean cp ± SD^a^	36.03 ± 1.49	35.58 ± 1.92	35.49 ± 1.35	32.68 ± 1.50	35.13 ± 1.24	33.46 ± 2.06	24.05 ± 1.98	32.87 ± 0.64	23.15 ± 1.45	24.10 ± 1.43	17.86 ± 0.70

^a^Mean crossing points (cp) ± SD were calculated using GD 15.5, 16.5, and 17.5 mouse fetal lungs. The values are estimations of the relative abundance of the indicated mRNAs (n = 16-20 pools). Samples in which no expression was detected were not included. The lower value, the higher mRNA abundance.

*Crh *mRNA levels were higher in fetal lung samples than in other tissues, including total brain (Figure 1A). In addition, a trend in increase in *Crh *mRNA levels was observed according to gestation time (p = 0.053). For *Crhbp *mRNA levels (Figure 1B), a significant interaction between time and sex (p = 0.019), as well as a significant impact of sex (p = 0.017), were observed. Moreover, expression levels tend to decrease according to gestational age. A high *Crhbp *mRNA level was detected in brain, which is recognized as the major expression site of this gene [33]. Very low levels of *Crhr1 *mRNA were observed in several fetal lung samples (Figure 1C; Table 2), while no expression was detected in the others. For comparison, brain and spleen expressed *Crhr1 *mRNA at relatively high levels, and no expression was detected in adult lung and adrenal gland. *Crhr2b *expression in fetal lung samples showed more interindividual variability than the other considered genes (Figure 1D), but no significant effect of time or sex was observed. A higher mRNA level was detected in adult lung than in the other control tissues. *Pomc *mRNA levels were similar between fetal lungs and adult tissues, except for adrenal gland in which expression level was very low (Figure 1E). *Mc2r *mRNA was detected in fetal lungs and was found to be expressed at much higher levels in two GD 15.5 samples (one female pool and one male pool) (Figure 1F). These two samples were previously shown to express high levels of genes involved in the adrenal glucocorticoid synthesis pathway (samples 70 F and 71 M in [22]). As expected, the expression level of *Mc2r *in adrenal gland was very high. No significant effect of time or sex was observed for *Pomc *and *Mc2r*. Gene expression of melanocortin receptor associated protein (MRAP), which is important in MC2R signaling [34], was also detected in fetal lung samples (Table 2 and data not shown). High *Nr3c1 *mRNA levels were detected in fetal lungs (Table 2) and a significant increase in expression was observed over time (about 2-fold between GD 15.5 and 17.5, p = 0.0003) (Figure 1G). Among the analyzed mature tissues, the highest *Nr3c1 *expression level was observed in the lung.

### Expression sites of HPA axis-related genes in male and female fetal mouse lungs

**Figure 2:**
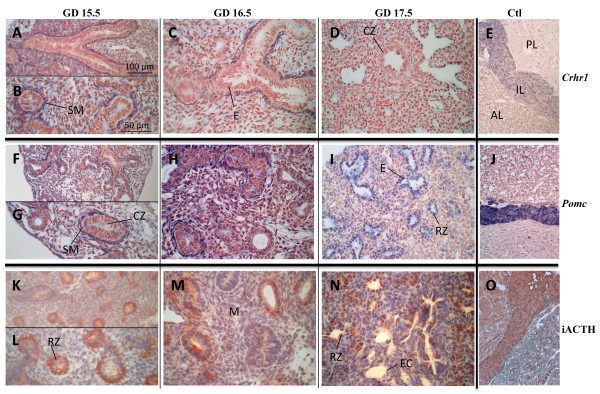
**Distribution of *Crhr1 *mRNA, *Pomc *mRNA, and immunoreactive (i) ACTH protein in mouse fetal lungs**. Tissue sections are from GD 15.5 (A, B, F, G, K and L), 16.5 (C, H and M) or 17.5 (D, I and N). Adult mouse pituitary sections are used as positive control (E, J and O). *In situ *hybridization was performed using anti-sense probes (A to J, positive signal is blue), while immunohistochemistry was performed using an anti-iACTH polyclonal antibody (K to O, positive signal is red). No staining was observed using corresponding sense probes (negative controls, data not shown). On GD 15.5, expression of *Crhr1 *(A and B) and *Pomc *(F and G) was observed in cells surrounding airways and a change in expression sites toward distal epithelial cells was observed according to developmental time. On GD 15.5 (K and L), 16.5 (M), and 17.5 (N), iACTH was detected in epithelial cells. Scale bars, 100 μm (A, E, F, J, K and O) or 50 μm (B to D, G to I and L to N). **AL**, anterior lobe; **CZ**, conducting zone; **E**, epithelial cells; **EC**, endothelial cells; **IL**, intermediary lobe; **M**, mesenchyme; **PL**, posterior lobe; **RZ**, respiratory zone; **SM**, smooth muscle.

**Figure 3:**
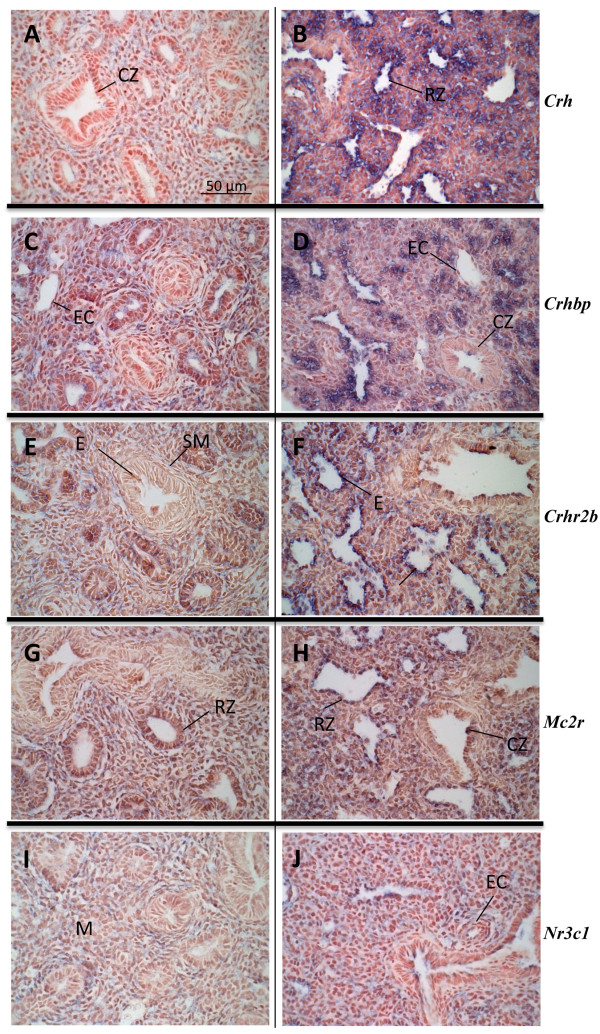
**Distribution of *Crh*, *Crhbp*, *Crhr2b*, *Mc2r*, and *Nr3c1 *mRNAs in mouse fetal lungs**. Mouse tissue sections are from GD 15.5 (A, C, E, G and I) or 17.5 (B, D, F, H and J). *In situ *hybridization was performed using anti-sense probes (A to J, positive signal is blue). No staining was observed using corresponding sense probes (negative controls, data not shown). Results obtained on GD 16.5 (data not shown) are similar to those obtained on GD 15.5 for all the genes. A change in expression sites between GD 15.5-16.5 *versus *17.5 was observed. Expression of mRNAs encoding for all the analyzed genes was observed in epithelial cells of distal zones on GD 17.5. Scale bars, 50 μm (A to J). **CZ**, conducting zone; **E**, epithelial cells; **EC**, endothelial cell; **M**, mesenchyme; **RZ**, respiratory zone; **SM**, smooth muscle.

*In situ *hybridization was performed on male and female fetal mouse lungs from GD 15.5, 16.5, and 17.5 to localize expression sites of *Crh*, *Crhbp*, *Crhr1*, *Crhr2b*, *Pomc*, *Mc2r*, and *Nr3c1 *(Figures 2, 3, and data not shown). In addition, iACTH was detected by immunohistochemistry (Figure 2).

*Crhr1 *and *Pomc *mRNAs were localized on GD 15.5 and 16.5 in cells surrounding epithelial cells of conducting zones (Figure 2A-C, F-H), whereas iACTH protein was detected in epithelial cells (Figure 2K-M). On GD 17.5, *Pomc *and iACTH signals were strong in epithelial cells of distal zones (Figure 2I and 2N). Adult pituitary gland was used as positive control and, as expected, *Crhr1*, *Pomc*, and iACTH were localized in intermediate and anterior lobes (Figure 2E J, and 2O) [35].

For *Crh*, *Crhbp*, *Crhr2b*, *Mc2r*, and *Nr3c1 *mRNAs, positive signals were widely distributed on GD 15.5 (Figure 3A, C, E, G, and 3I) and 16.5 (data not shown). However, no signal was observed in epithelial cells of conducting zones, except for a few cells. For these five genes, as for *Crhr1 *and *Pomc*, an important change in expression sites is observed when tissues originating from GD 15.5-16.5 and GD 17.5 are compared, the distal epithelium being the major site of expression on GD 17.5 (Figure 3B, D, F, H, and 3J). *Nr3c1 *mRNA was also detected in mesenchymal cells, although at lower levels than in epithelial cells. For every gene studied, no sex difference was observed in mRNA expression sites.

### Expression levels of HPA axis-related genes in primary cell cultures derived from fetal mouse lungs

**Figure 4:**
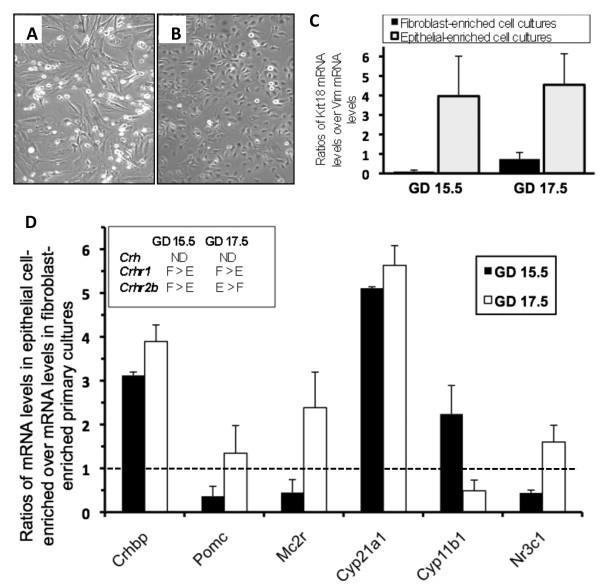
**Relative mRNA expression levels of HPA axis-related genes in differential cell cultures derived from fetal mouse lungs**. *Crh*, *Crhbp*, *Crhr1*, *Crhr2b*, *Pomc*, *Mc2r*, *Cyp21a1*, *Cyp11b1*, and *Nr3c1 *were quantified by QPCR in fibroblast-enriched and in epithelial cell-enriched primary cultures originating from GD 15.5 and 17.5 fetal mouse lungs. Representative cultures of mesenchymal cells (A) and epithelial cells (B) are presented. Expression ratios of *Krt18 *(cytokeratin 18; epithelial cell marker) on *Vim *(vimentin; mesenchymal cell marker) were assessed to confirm cell enrichment (C). For each studied gene, ratios of expression levels in epithelial cells over expression levels in mesenchymal cells are presented (D). Genes for which all or some of the samples were negative are presented in the inset. Values that are over the dashed line represent higher expression level in epithelial cell-enriched cultures. **ND**, not detected; **E**, epithelial cell-enriched cultures; **F**, fibroblast-enriched cultures.

Next, we determined whether the transition in gene expression sites from GD 15.5 to 17.5 can be observed in cell cultures. Levels of *Crh*, *Crhbp*, *Crhr1*, *Crhr2b*, *Pomc*, *Mc2r*, and *Nr3c1 *mRNAs were compared between fibroblast-enriched cell cultures, epithelial cell-enriched cell cultures, and total lungs. Fetal sex was not considered in these experiments because only *Crhbp *showed a sex difference in expression by QPCR in whole lungs, whereas no sex difference was observed for any of these genes by *in situ *hybridization. Messenger RNA levels of *Krt18 *and *Vim*, which are epithelial and mesenchymal markers, respectively, confirmed cell enrichments (Figure 4C). For each analyzed gene, ratios corresponding to mRNA levels in epithelial cell-enriched cultures over those in fibroblast-enriched cultures are presented in Figure 4D. Interestingly, *Crh *mRNA was not or barely detected in cell cultures. On GD 15.5 and 17.5, *Crhr1 *mRNA was detected in mesenchymal cells, whereas the transcript was not or barely detected in epithelial cells. On GD 15.5, *Crhr2b *expression level was higher in mesenchymal cells than in epithelial cells, while on GD 17.5 the opposite situation was observed. *Crhr2b *mRNA was not detected in all samples. *Crhr1 *and *Crhr2b *mRNA levels were lower in cell cultures than in whole fetal lungs, similarly to *Crh *(data not shown). The expression of *Crhbp *was higher in epithelial cells than in fibroblasts on GD 15.5 and 17.5, while *Pomc*, *Mc2r*, and *Nr3c1 *are preferentially expressed in fibroblasts on GD 15.5 and in epithelial cells on GD 17.5 (Figure 4D). Expression levels of these last four genes were similar between cell cultures and total lung pools (data not shown).

### Expression of Cyp21a1 and Cyp11b1 in fetal mouse lungs and fetal lung primary cell cultures

*Cyp21a1 *and *Cyp11b1 *encode the last two steroidogenic enzymes involved in *de novo *corticosterone synthesis [36,37]. *Cyp11b1 *expression was previously observed on GD 15.5, but not on GD 16.5, 17.5, and 18.5 in the fetal lung [22]. There was no available data on expression of *Cyp21a1 *after GD 15.5. We detected *Cyp21a1 *mRNA in fetal lung pools and in lung explants obtained on GD 17.5 (data not shown). Interestingly, *Cyp21a1 *mRNA was detected at much higher levels in primary cell cultures than in non-incubated whole lung samples and lung explants. Indeed, *Cyp21a1 *mRNA levels were ~15 and ~40 fold higher in fibroblast- and in epithelial cell-enriched cultures, respectively, than in lung explants (data not shown), and were higher in epithelial fractions than in fibroblast fractions on both GD 15.5 and 17.5 (Figure 4D). In agreement with previous data, *Cyp11b1 *mRNA was not detected in lung explants obtained on GD 17.5 in the present study. However, *Cyp11b1 *mRNA was surprisingly detected at relatively high levels in cell cultures, as *Cyp21a1.*

The potential effect of reciprocal exposure of epithelial- and mesenchymal-enriched cell cultures to their respective secreted factors on gene expression of *Crh*, *Crhr1*, *Crhr2β*, *Cyp21a1*, and *Cyp11b1 *was addressed. Target gene mRNA levels were quantified in epithelial- and mesenchymal-enriched cells after co-culture using porous filters (0.1 μm) during 96 h (n = 16 fetal lungs from 3 litters). *Crhr2β *mRNA level was 4-fold higher in co-cultured fibroblasts than in fibroblasts cultured alone. For the other analyzed genes, results obtained from co-cultured cells were similar to those from separated cells (data not shown).

### Incubation of fetal mouse lung explants in the presence of CRH or ACTH, and determination of glucocorticoid formation

**Figure 5:**
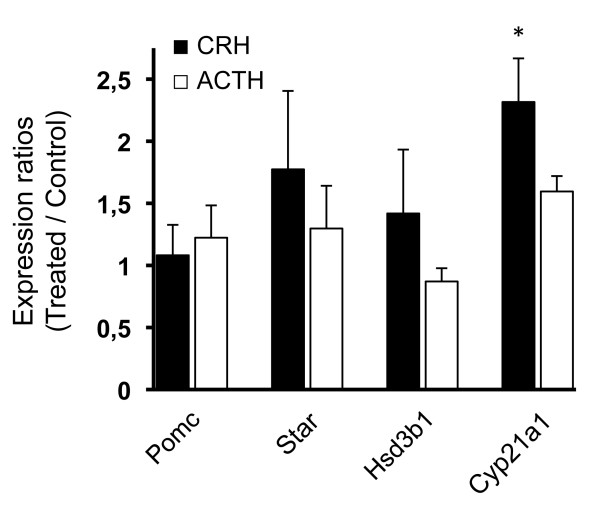
**Effects of exogenous CRH or ACTH on expression levels of *Pomc*, *Star*, *Hsd3b1*, and *Cyp21a1 *in mouse fetal lung explants**. Fetal lungs were harvested on GD 17.5 and incubated 8h in the presence of 10^-7 ^M CRH or 10^-7 ^M ACTH. Messenger RNA levels of *Pomc*, *Star*, *Hsd3b1*, and *Cyp21a1 *were measured by QPCR in control and treated lungs. Data are presented as ratios of treated samples over control samples ± SEM. Experiments were performed with 1 to 3 fetal lungs per condition per litter (n = 4 litters), and statistical analysis was performed using one-way ANOVA with randomized-block design (samples within each litter were matched). *, *P *< 0.05

GD 15.5 and 17.5 lung explants were incubated in the presence of CRH or ACTH to evaluate the potential modulatory roles of these hormones on mRNA levels of *Pomc*, *Star*, *Hsd3b1*, *Cyp21a1*, and *Cyp11b1*. The fetal sex was not considered for the reasons exposed above. No effect of CRH or ACTH was observed on GD 15.5 on the expression level of any of the studied genes (data not shown). On GD 17.5, a significant stimulatory effect of CRH (n = 4, ANOVA, p = 0.0307; Tukey's test Ctl vs CRH, p < 0.05) was observed on *Cyp21a1 *mRNA levels (Figure 5). No significant effect was observed on *Pomc*, *Star*, and *Hsd3b1 *expression. To determine whether the CRH-induced increase in *Cyp21a1 *expression leads to increased enzymatic activity, we measured the level of newly-formed tritiated deoxycorticosterone in control and CRH-treated fetal lung explants isolated on GD17.5 using tritiated progesterone as substrate. Unlabeled deoxycorticosterone was added to reduce metabolization of newly-formed tritiated deoxycorticosterone. Accumulation of tritiated deoxycorticosterone was detected in all samples, and a non-statistically significant 25% increase of deoxycorticosterone production was observed in CRH-treated samples compared to controls (paired t-test, p = 0.36) (data not shown). No corticosterone accumulation was detected.

## Discussion

We report expression of HPA axis-related genes in the fetal lung during late gestation, a period that includes the surge of surfactant synthesis occurring on GD 17 in the mouse. More precisely, we present expression profiles of *Crh*, *Crhr1*, *Crhr2b*, *Crhbp*, *Pomc*, *Mc2r*, and *Nr3c1 *and we also report marked co-expression of *Mc2r *with genes encoding for glucocorticoid synthesizing enzymes from cholesterol on GD 15.5. We show that *Crhr1 *and *Pomc *mRNAs are localized in cells surrounding proximal epithelia where iACTH is detected on GD 15.5 and 16.5, and that a major switch in expression sites toward epithelial cells of distal zones occurred between GD 15.5 and 17.5 for all the studied genes. In addition, a significant CRH-modulated increase in *Cyp21a1 *expression was observed in fetal lung explants isolated on GD 17.5 as well as a deoxycorticosterone production by fetal lung explants. Even though our data do not suggest a functional HPA axis regulating the previously reported pathway of glucocorticoid synthesis on GD 15, they support roles for HPA axis-related components, as well as autocrine/paracrine actions of CRH and ACTH, in developing lungs.

In several species, a local metabolism of glucocorticoids through the action of 11β-hydroxysteroid dehydrogenases (11βHSDs) was shown in the developing lung where it leads to a net increased formation of active glucocorticoids by the predominant 11βHSD type 1 activity [38-42]. It was also reported that the fetal mouse lung expresses all the genes involved in glucocorticoid synthesis from cholesterol [22]. High expression of the latter genes was observed in several litters on GD 15 but only in male or female lung pools according to the analyzed litter, and this occurred before an important rise in *Hsd11b1 *expression [22]. Interestingly, in the present study, *Mc2r *was highly expressed in the same pools where the genes involved in glucocorticoid synthesis from cholesterol showed high expression levels, thus suggesting a role for *Mc2r *in the control of the "adrenal" pathway of glucocorticoid synthesis in the fetal lung. Accordingly, expression of *Mrap *was detected in fetal lung samples, the expression of this gene being necessary for MC2R signaling [34]. We localized a weak *Mc2r *mRNA signal in distal epithelial cells and in the mesenchyme on GD 15.5, while staining in epithelial cells was more intense on GD 17.5. MC2R was previously detected by immunohistochemistry in fetal mouse lungs from GD 11.5 to 14.5 in the bronchial epithelium and in the mesenchyme [15]. Taken together, these observations suggest that the site of *Mc2r *expression varies according to gestational time. Expression of *Mc2r *has been shown to be regulated temporally in the fetal testis [43], as in the fetal lung.

In the present study, incubation of lung explants isolated on GD 15.5 with CRH or ACTH did not up-regulate the genes involved in the "adrenal" pathway of glucocorticoid synthesis. A postulated narrow window of CRH and/or ACTH receptivity may explain the absence of stimulation. Indeed, the fast progression of tissue development observed in the mouse where gestation is completed after only 19 days adds to the difficulty to characterize transitory events occurring within a very narrow developmental time window. Alternatively, it is not excluded that the previously reported transient co-expression of the five genes of the "adrenal" pathway may not be dependant on the presence of CRH and/or ACTH. Nevertheless, a significant increase in *Cyp21a1 *mRNA expression was observed in lung explants isolated on GD 17.5 following incubation with CRH, along with a 25% increased deoxycorticosterone production (p = 0.36). The high expression levels previously observed on GD 15 for the genes of the cascade were not reached in both control and stimulated lung explants in the present study, suggesting that the absent or modest activation of expression reported here for these genes is not a consequence of a saturated system caused by endogenous CRH and ACTH production.

After GD 15.5, 21-hydroxylase may not be involved in corticosterone synthesis since *Cyp11b1 *mRNA is neither expressed in intact tissue [22] nor stimulated following CRH or ACTH incubations in lung explants (Figure 5). A common observation to both adult mouse lung [21] and fetal lung after GD 15.5 is that expression of *Cyp11a1 *and *Cyp21a1*, but not that of *Cyp11b1*, occur. Interestingly, the immediate corticosterone precursor, deoxycorticosterone, produced from progesterone by 21-hydroxylase, was shown to have a low glucocorticoid effect [44,45]. In the present study, steroidogenic activity experiments showed deoxycorticosterone production by fetal lungs through 21-hydroxylase activity, as also reported in [46]. In addition, based on the reported affinity of deoxycorticosterone for the mineralocorticoid receptor [47] and on the very low aldosterone synthase mRNA level in fetal lungs ([22] and data not shown), one would suggest that this steroid could have also mineralocorticoid effects in the lung.

Incubation of GD 17.5 lung explants in the presence of ACTH led to a non-significant 1.5-fold increase in *Cyp21a1 *expression, and the stimulatory effect of CRH on *Cyp21a1 *did not involve an increase in *Pomc *mRNA expression. Therefore, if CRH acted through ACTH, it would rather be at another regulatory level, such as prohormone processing or hormone release. A similar slight but not statistically significant effect of ACTH was observed on the capability of thymic epithelial cells to stimulate a glucocorticoid-regulated reporter system [21]. Further studies are needed to clarify whether a local HPA axis-like regulatory pathway is functional in the fetal lung as it is the case in the adult skin, where glucocorticoid production has been shown to be regulated by a local cascade of CRH and ACTH production and signaling [19].

In epithelial- and mesenchymal-enriched primary cell cultures, *Crh*, *Crhr1*, and *Crhr2b *were not or barely detected whereas *Cyp11b1 *and *Cyp21a1 *were detected at relatively high levels. A different situation was observed in total lungs for these five genes. Such noticeable differences in expression levels between primary cell cultures and total lungs were not observed for the other studied genes. These discrepancies should not arise from cell enrichment but rather from dysregulation of regulatory pathways that are active in the whole tissue because: *Crh *expression was undetectable in both epithelial-enriched and mesenchymal-enriched cell cultures but was clearly detected in vivo by in situ hybridization in cell types that are represented in cell cultures, and; mRNA levels of *Cyp11b1 *were elevated in both epithelial-enriched and mesenchymal-enriched cell cultures in contrast to whole lungs where only barely detectable levels were detected. Accordingly, cell-cell communication between mesenchymal and epithelial cells was reported in the developing lung [48], and may be involved in the in vivo regulation of the above-mentioned genes. Results from our co-culture experiments suggest that such a potential regulation is not explained alone by secreted factors from epithelial and mesenchymal cells. A similar situation was also observed in the lung where glucocorticoids had differential effects on the expression of *Nr3c1 *in fetal lung primary cell cultures compared to whole fetal lung tissues in the rat [49].

Our data suggest paracrine and/or autocrine regulation and action of CRH and POMC-related peptide(s) in the developing lung. However, an effect of circulating ligands on specific CRH and ACTH receptors expressed in the lung is not excluded. Noteworthily, changes in expression sites of the studied receptors also suggest different functions according to the developmental status of the tissue.

*Crh *mRNA was detected at higher levels in fetal lung samples than in control tissues, including the adult lung, which is consistent with a specific role for CRH in lung development (Figure 1). Accordingly, detection of *Crh *mRNA by *in situ *hybridization was reported on GD 12.5 in the mouse lung around branching bronchioles with an increase until GD 15.5, followed by a constant expression level until GD 17.5, while no signal was detected on GD 18.5 and postnatal day 1 [11]. However, the change in *Crh *expression sites that we present here was not reported in that study. Interestingly, in a previous microarray analysis in mouse fetal lungs, *Crh *expression was significantly higher in lungs from males exposed to the anti-androgen flutamide compared to control males (~50% increase, p < 0.05), suggesting an inhibitory effect of endogenous androgens on *Crh *expression [27]. We observed by QPCR that *Crh *mRNA levels tended to increase between GD 15.5 and 17.5, while levels of *Crhbp *tended to decrease. This is suggestive of an increased CRH bioactivity considering the inhibitory action of CRHBP on CRH signaling [33]. *Crhbp *was the only gene showing a significant sex difference in expression in the present study. Interestingly, a sex dimorphism in *Crhbp *expression was reported in the mouse pituitary gland [50]. *Crhr1 *mRNA was detected at very low levels in fetal lungs. However, the fact that its expression is limited to a low proportion of cells may explain the low mRNA levels measured from total lung extracts. Similarly to our *Crhr1 *expression data, an important interindividual variability in *CRHR1 *mRNA levels was observed in developing fetal baboon lungs, with detection in about 30% of samples on GD 125 (term ~180 days) [14].

Interestingly, *Crhr1 *and *Pomc *mRNAs are co-localized on GD 15.5 and 16.5 in cells surrounding the proximal epithelium. In contrast, this staining pattern was not observed on GD 17.5. These results suggest that CRHR1 signaling could lead to ACTH production in a developmental time-specific manner. However, the question whether MC2R ligand is produced in the lung or is imported from the circulation still remains. The detection of immunoreactive ACTH in cells adjacent to those expressing *Pomc *mRNA (GD 15.5-16.5) and in *Pomc *mRNA-positive cells (GD 17.5) suggests a paracrine/autocrine action of ACTH. Gene expression of several prohormone convertases [51], namely FURIN, endoprotease PACE4, proprotein convertase subtilisin/kexin (PCSK) type 5, and PCSK7 were detected in the developing mouse lung at GD 17.0 and 18.0 by DNA microarrays [27]. *Pc1/3*, which encodes the prohormone convertase 1/3 that is associated to ACTH production in the pituitary, was not detected in this gene profiling experiment. However, FURIN and PACE4 have been shown to yield ACTH from POMC [52].

This report shows a transition in expression sites between GD 15.5 and 17.5 for all the studied genes (Figures 2 and 3). These developmental time-specific expression profiles are partly supported by QPCR data obtained from mesenchymal- and epithelial-enriched primary cell cultures (Figure 4). On GD 17.5, expression of every gene was mainly localized in the distal epithelium. This pattern of expression is interesting in the context of lung maturation since the surge of surfactant production occurs on GD 17 in some epithelial cells of this epithelium. Thus, a role for CRH/ACTH in maturation and/or development of the distal epithelium is envisaged.

## Conclusions

Temporal and spatial expression patterns of HPA axis-related genes in fetal lungs during late gestation suggest local roles for CRH and POMC/ACTH in lung development. Our data are likely to lead to valuable insights in relation to lung diseases originating from lung immaturity.

## Competing interests

The authors declare that they have no competing interests.

## Authors' contributions

MS participated in the design of the study, carried out QPCR experiments, participated in tissue collection, in cell culture preparation, and in experiments involving fetal lung explants, and drafted the manuscript; MC carried out ISH experiments, participated in tissue collection, in cell culture preparation and in experiments involving fetal lung explants, and helped to draft the manuscript; PRP participated in tissue collection, in the design of the study, and helped to draft the manuscript; YT participated in the design of the study and in its coordination, and helped to draft the manuscript. All authors read and approved the final manuscript.
